# Application of Neural Network and Cluster Analyses to Differentiate TCM Patterns in Patients With Breast Cancer

**DOI:** 10.3389/fphar.2020.00670

**Published:** 2020-05-08

**Authors:** Wei-Te Huang, Hao-Hsiu Hung, Yi-Wei Kao, Shi-Chen Ou, Yu-Chuan Lin, Wei-Zen Cheng, Zi-Rong Yen, Jian Li, Mingchih Chen, Ben-Chang Shia, Sheng-Teng Huang

**Affiliations:** ^1^ Department of Chinese Medicine, China Medical University Hospital, Taichung, Taiwan; ^2^ Graduate Institute of Business Administration, College of Management, Fu Jen Catholic University, New Taipei City, Taiwan; ^3^ Research Center of Big Data, College of Management, Taipei Medical University, Taipei, Taiwan; ^4^ Information Technology Office, China Medical University Hospital, Taichung, Taiwan; ^5^ College of Management, Taipei Medical University, Taipei, Taiwan; ^6^ Executive Master Program of Business Administration in Biotechnology, College of Management, Taipei Medical University, Taipei, Taiwan; ^7^ School of Chinese Medicine, China Medical University, Taichung, Taiwan; ^8^ Research Center for Traditional Chinese Medicine, Department of Medical Research, China Medical University Hospital, Taichung, Taiwan; ^9^ Chinese Medicine Research Center, China Medical University, Taichung, Taiwan; ^10^ Research Center for Chinese Herbal Medicine, China Medical University, Taichung, Taiwan; ^11^ Department of Chinese Medicine, An-Nan Hospital, China Medical University, Tainan, Taiwan

**Keywords:** traditional Chinese medicine, electronic medical records, breast cancer, neural network analysis, cluster analysis, pattern differentiation

## Abstract

**Background and Purpose:**

Pattern differentiation is a critical element of the prescription process for Traditional Chinese Medicine (TCM) practitioners. Application of advanced machine learning techniques will enhance the effectiveness of TCM in clinical practice. The aim of this study is to explore the relationships between clinical features and TCM patterns in breast cancer patients.

**Methods:**

The dataset of breast cancer patients receiving TCM treatment was recruited from a single medical center. We utilized a neural network model to standardize terminologies and address TCM pattern differentiation in breast cancer cases. Cluster analysis was applied to classify the clinical features in the breast cancer patient dataset. To evaluate the performance of the proposed method, we further compared the TCM patterns to therapeutic principles of Chinese herbal medication in Taiwan.

**Results:**

A total of 2,738 breast cancer cases were recruited and standardized. They were divided into 5 groups according to clinical features *via* cluster analysis. The pattern differentiation model revealed that liver-gallbladder dampness-heat was the primary TCM pattern identified in patients. The main therapeutic goals of the top 10 Chinese herbal medicines prescribed for breast cancer patients were to clear heat, drain dampness, and detoxify. These results demonstrated that the neural network successfully identified patterns from a dataset similar to the prescriptions of TCM clinical practitioners.

**Conclusion:**

This is the first study using machine-learning methodology to standardize and analyze TCM electronic medical records. The patterns revealed by the analyses were highly correlated with the therapeutic principles of TCM practitioners. Machine learning technology could assist TCM practitioners to comprehensively differentiate patterns and identify effective Chinese herbal medicine treatments in clinical practice.

## Introduction

Breast cancer is the most common cancer affecting the female population globally. As an adjunct for cancer treatments, complementary and alternative medicine (CAM) is an increasingly popular option sought by patients with breast cancer (Crocetti et al., 1998; Balneaves et al., 2006; Boon et al., 2007). Meanwhile, Traditional Chinese Medicine (TCM) is an important component of CAM, and is currently widely used by breast cancer patients in the ethnic Chinese population (Chen et al., 2008). Many patients seek TCM to resolve side effects including nausea and vomiting, fatigue, paresthesia, chronic pain, constipation, and anorexia which may result from standard Western medicine cancer treatments (Chung et al., 2016).

Despite the increased popularity of TCM, modernization in the field of TCM remains gradual (Ling and Xu, 2013). One particular limitation lies in the fact that the diagnostic and therapeutic systems of TCM depend heavily on the notion of pattern differentiation. The TCM pattern is a diagnostic summary of each individual based on four diagnostic methods: observation, listening, questioning, and pulse detection (World Health Organization. Regional Office for the Western, 2007). Until recently, inefficient data extraction methods have limited the development of automated TCM pattern differentiation. Furthermore, the combinational and highly individualized nature of TCM prescriptions in clinical practice create challenges for researchers to successfully execute randomized control trials to verify TCM theories.

In recent decades, access to electronic medical records (EMR) and advanced machine-learning techniques have enabled the development of computational methods to enhance the field of TCM. More specifically, researchers can now automate the data mining process through natural language processing and information extraction methods. A previous study has demonstrated a framework of automatic diagnosis of TCM by analyzing raw free-text clinical records (Wang et al., 2012).

Artificial neural networks (ANN) are non-linear models that have shown to be useful in elucidating the relationship between the input and output signals of a complex system (Zhang et al., 2018). In this study, we utilized DeepMedic software which incorporated TCM pattern data with ANN to differentiate the TCM patterns identified in individual breast cancer patients. A series of methods including cluster analysis were applied to analyze a dataset of EMR. The cluster analysis was also applied to evaluate the relationships between clinical features, referred to as symptoms and signs in TCM clinical practice, to distinguish TCM pattern differentiation. To evaluate the performance of the TCM pattern differentiation system developed for our study, we further compared the TCM patterns identified in each cluster subgroup with the top ten Chinese herbal prescriptions for Taiwanese breast cancer patients (Huang et al., 2017).

The aim of this study was to apply neural network analysis and cluster analysis to reveal patterns from an EMR dataset and to compare them with the prescriptions of TCM clinical practitioners for the treatment of patients with breast cancer in Taiwan.

## Materials and Methods

### Data Acquisitions

The EMR of breast cancer patients (ICD-9 174.0–174.9) having received TCM treatment between January 01, 2003 and June 15, 2018 were collected from the China Medical University Hospital (CMUH) database. The diagnoses were based on the International Classification of Diseases, Ninth Revision, Clinical Modification (ICD-9-CM). This study was approved by the Research Ethics Committee of China Medical University and Hospital, Taichung, Taiwan (CMUH107-REC2-023). All of the datasets analyzed were decoded so that the review board waived the requirement to sign informed consent from patients.

### DeepMedic Neural Network Analysis

In this study, we used the DeepMedic software to standardize the terminologies of TCM, and to summarize the most likely TCM pattern in each case. The standardization process aimed to unify the polysemous or synonymous vocabulary used in the TCM diagnostic system to facilitate the neural network analysis. The standardization process was accomplished by modifying symptom vocabulary to match the thesaurus within the DeepMedic software, which contains over 20,000 symptom terminologies. Respective standard nomenclatures were applied in the standardization process of syndrome elements, TCM patterns, and treatment modalities. The DeepMedic software can convert TCM patterns into several codes, and label the standard TCM terminologies. For each case being analyzed as input, the specific TCM pattern was identified by determining the higher-weighted code of symptoms and signs. A forward and backward propagation of the neural network, consisting of several hidden layers, was used to calculate the weightings of each code. The weighting of each pattern was based on different symptoms and signs, calculated by using the well-known heuristic equation, Term-Frequency-Inverse Document Frequency (TF-IDF), with some modifications.

TF = (the frequencies of symptom A in code B/code)

$$Term\;frequency=f_{t,d}∕\sum_{t^{\prime }\in d}f_{t^{\prime },d}$$

$$Inverse\;document\;frequency\;smooth=\log\left(1+\text{N}∕{\text{n}}_{\text{t}}\right)$$

The efficacies, as well as the details of related methods, have been demonstrated in our previous study (Lin et al., 2019). The website accessing the demo version of DeepMedic software can be found at: http://bigdata-demo.deepmedic.cn/.

### Cluster Analysis

In statistical methodologies, the purpose of cluster analysis is to group the classification objects according to the characteristics of the particular dataset. Study objects classified to the same group have similar characteristics, while those classified to different groups indicate that there are considerable differences in the characteristics. We used K-means cluster to divide data into groups, and the number of clusters was determined by using the smallest total within the sum of squares.

### Key Performance Indicators (KPI)

Each variable in the dataset of this study was recorded by binary classification of “yes/no”. Additionally, more even variables are more effective at finding similarity between each cluster. Therefore, we calculated the mean and standard deviation from all variables according to the concept of coefficient of variation. The KPI obtained from dividing the standard deviation by the mean is used for selecting variables. The statistical formula is shown below. The higher value of this statistic represents more even variables. In order to find the optimal KPI, we limited the capture frequency of the variable to more than 5%. Starting from the minimum KPI, we increased the interval by 0.01 to find the best one.

$$KPI_{item}=\frac{s}{\bar{x}}=\frac{\sqrt{\frac{item_{yes}}{item_{all}}\times \frac{item_{no}}{item_{all}}}}{\frac{item_{yes}}{item_{all}}}=\frac{\sqrt{item_{yes}\times item_{no}}}{item_{yes}}=\sqrt{\frac{item_{no}}{item_{yes}}}$$

### The Analysis of Symptoms and Signs in Cluster Model

If there were no statistically significant differences and greater than 5% frequency of a variable among clusters, this variable would be determined as a primary feature (PF). Additionally, symptoms that had significant differences in frequency but similar rankings, where the difference between the highest and the lowest ranking was not more than 10 and all frequencies of this variable in each cluster were more than 5% among clusters, were considered the primary features of breast cancer cases, since these symptoms had similar importance in each cluster. When the cluster analytical result of KPI has the most number of primary features, it will be defined as the best KPI.

A symptom is defined as a subjective experience of a disease or physical ailment reported by a patient, while a sign is defined as any abnormal indication of disease that is identified by TCM practitioners (Dodd et al., 2001). Pulse and tongue inspections are the primary diagnostic methods applied by TCM practitioners to collect the data of clinical signs. Despite the correlation between symptoms and signs, the data collection methodologies are different; therefore, we separately collected and analyzed data of symptoms and three types of signs for subsequent TCM pattern differentiation.

Clinical signs including tongue appearance, tongue coating, and pulse were analyzed individually due to variables. The symptoms and signs were ranked according to the frequency of concurrent events. To make the high-ranking symptom and sign variables more representative, we excluded variables with a frequency of less than 5%, and the remaining variables were regarded as secondary features (SF) in each cluster.

### TCM Pattern Identification With Various PF and SF

From the previous analysis, we obtained the PF and SF of each cluster in the cluster analysis with the best KPI. Each SF had different chances in the cluster due to differing frequencies. In order to analyze various possibilities, we disassembled the SF in a cluster and combined them into “Sx_n”. Where “x” was the number of a cluster, and “n” was the top number of symptoms of the SF. For example, S1_5 represented the top five symptoms of the SF in cluster 1 and its frequency was judged by the fifth symptom. Finally, these were combined with the PF as “P + Sx_n”. DeepMedic software was applied to objectively analyze the general TCM pattern of all combinations. We counted the number of various types of patterns and weighted each pattern with the frequency of the last symptom in each combination to calculate the percentage of this pattern occurring in the cluster. The percentage of a pattern equal to the average frequency of a pattern was divided by the sum of average frequency of all patterns. The statistical formula is shown below.

$$\begin{array}{l} Percentage_{ij}=\frac{f_{ij}}{F_{i}}\\ F_{i}=Sum\;of\;average\;frequency\;of\;patterns\;in\;cluster\;i\\ f_{ij}=Average\;frequency\;of\;pattern\;j\;in\;cluster\;i\\ i=1,\;2,\;\ldots ,\;5\\ j=1,\;2,\;\ldots ,\;number\;of\;patterns\;in\;cluster\;i \end{array}$$

### Chinese Herbal Prescriptions in Breast Cancer Patients

TCM herbs were classified into several categories based on their usage. To prove that the study objects are compatible with the clinical prescriptions, we analyzed the top 10 single herbs and formulas prescribed by clinical TCM practitioners in Taiwan (Huang et al., 2017). To compare the usage in frequency and dose of each herb and formula, we ranked these medications according to the value obtained by the number of person-days multiplied by average daily dose.

**Figure 1:**
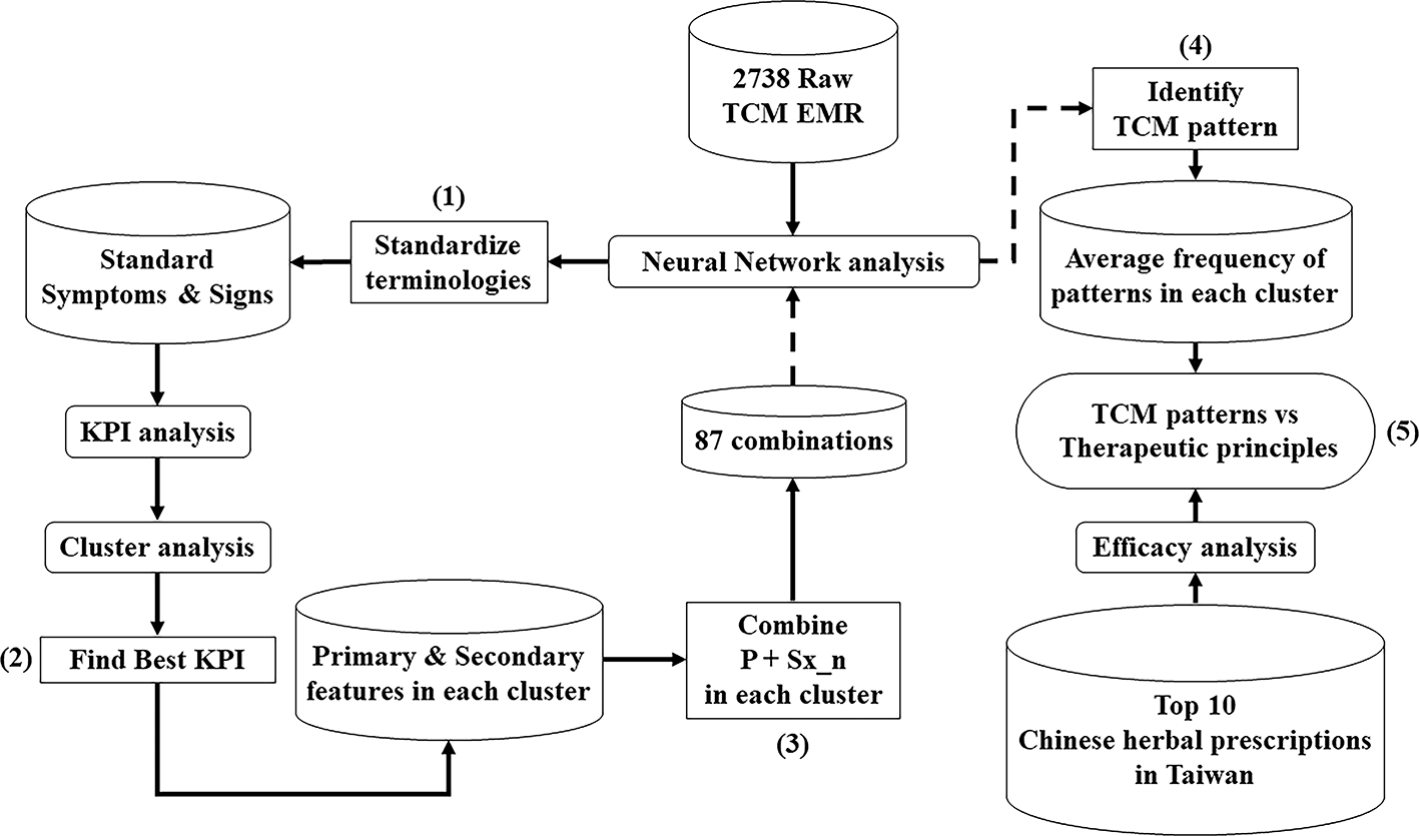
The analytical architecture of TCM EMR in the patients with breast cancer. The workflow (1) ~ (5) describes the process of multiple analyses to classify TCM clinical features and patterns of breast cancer patients, compared with therapeutic principles of Chinese herbal prescriptions by TCM practitioners in Taiwan.

Overall, the architecture (see **Figure 1**) of this study is primarily composed of five steps, as shown below.

1. Standardize the terminologies of TCM.
2. Find the best KPI to indicate that cluster analytical result has the most number of primary features.
3. Combine primary features and secondary features into different arrangements in each cluster.
4. Identify TCM patterns of each combination in each cluster through machine-learning confirmation.
5. Compare the similarity between TCM patterns in each cluster and the therapeutic principles of Top 10 Chinese herbal prescriptions in Taiwan.

## Results

### Data Extraction

**Figure 2:**
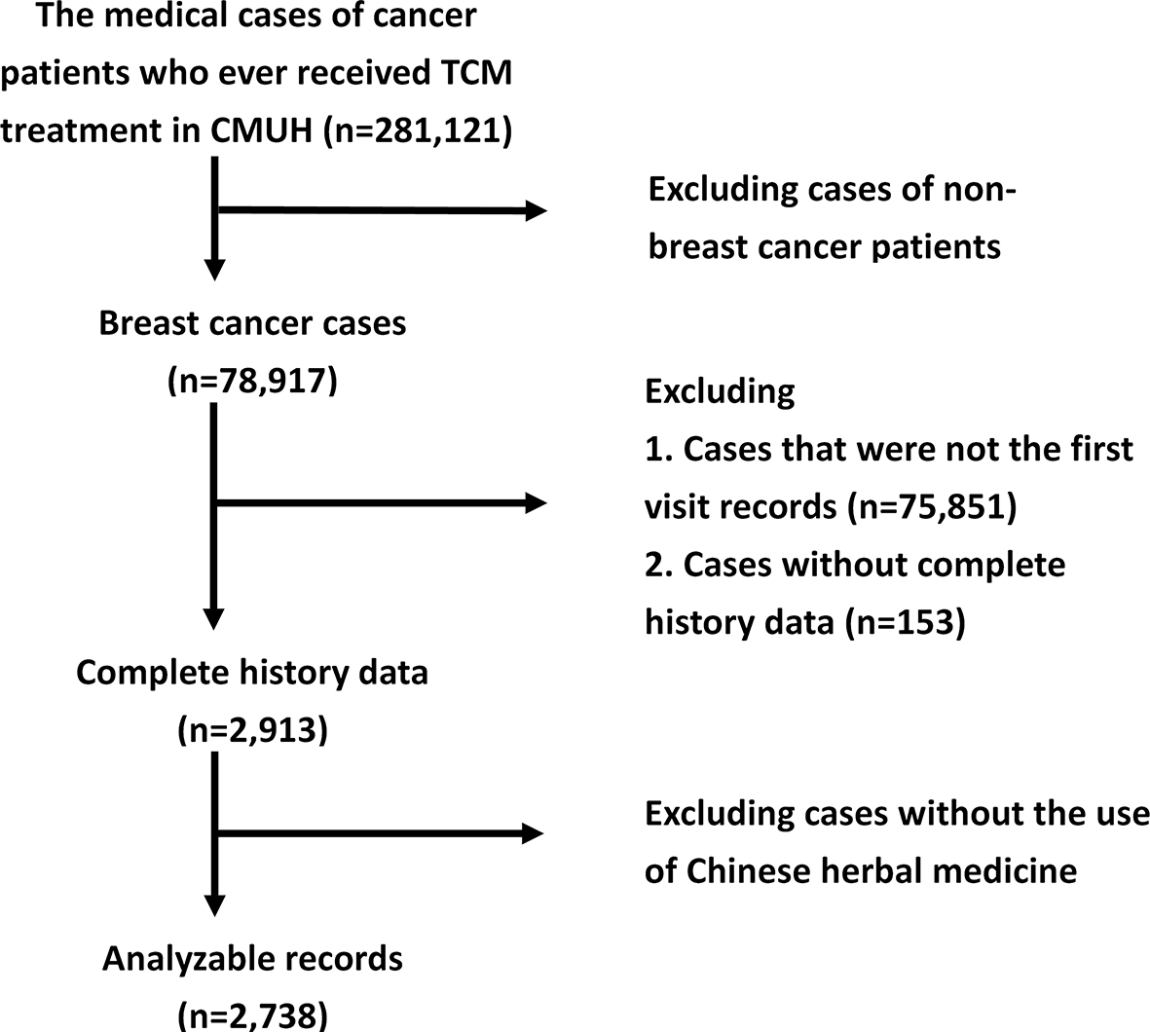
Flow chart of study cases in CMUH.

We selected only the initial visit records of individual patients, and excluded the remaining follow-up records, which contained incomplete data. All of these records must have included patient's gender, age, and details concerning symptoms and signs. A total of 78,917 breast cancer patients' records were recruited, including 2,913 complete initial visit records, of which 2,738 cases contained records of the specific herbs and formulas prescribed. The flowchart of our data acquisition is shown in **Figure 2**.

### The Standardization of Clinical Features

In the 2,738 analyzable records, the top twenty symptoms in frequency included “insomnia”, “dry mouth”, “lack of strength”, “dizziness”, “loss of appetite”, “abdominal distention”, “profuse dreaming”, “bitter taste of mouth”, “lumbago”, “back pain”, “afraid of cold”, “loose stool”, “headache”, “nausea”, “absence of thirst”, “cough”, “acid regurgitation”, “soreness”, “nocturia”, and “dry eyes”. The top five tongue appearances included “pale red tongue”, “red tongue”, “teeth-marked tongue”, “dark red tongue”, and “dry tongue”. The top five tongue coatings included “white coating”, “thin coating”, “thin white tongue”, “slimy coating”, and “thick coating”. The top five pulses included “string-like pulse”, “slippery pulse”, “fine pulse”, “weak pulse”, and “sunken pulse”. The ranking and frequency of each symptom and sign are listed in **Table 1**.

**Table 1:** The top 20 symptoms and the top five signs in breast cancer patients.

Rank	Symptom n(%)	Rank	Sign n(%)
1	Insomnia 1060 (38.7%)		**Tongue appearance**
2	Dry mouth 863 (31.5%)	1	Pale red tongue 516 (18.8%)
3	Lack of strength 467 (17.1%)	2	Red tongue 460 (16.8%)
4	Dizziness 395 (14.4%)	3	Teeth-marked tongue 241 (8.8%)
5	Loss of appetite 377 (13.8%)	4	Dark red tongue 137 (5.0%)
6	Abdominal distention 273 (10.0%)	5	Dry tongue 120 (4.4%)
7	Profuse dreaming 266 (9.7%)		
8	Bitter taste of mouth 234 (8.5%)		**Tongue coating**
9	Lumbago 231 (8.4%)	1	White coating 1163 (42.5%)
10	Back pain 199 (7.3%)	2	Thin coating 804 (29.4%)
11	Afraid of cold 194 (7.1%)	3	Thin and white coating 634 (23.2%)
12	Loose stool 192 (7.0%)	4	Slimy coating 368 (13.4%)
13	Headache 189 (6.9%)	5	Thick coating 213 (7.8%)
14	Nausea 181 (6.6%)		
15	Absence of thirst 175 (6.4%)		**Pulse**
16	Cough 175 (6.4%)	1	String-like pulse 865 (31.6%)
17	Acid regurgitation 170 (6.2%)	2	Slippery pulse 520 (19.0%)
18	Soreness 143 (5.2%)	3	Fine pulse 443 (16.2%)
19	Nocturia 139 (5.1%)	4	Weak pulse 369 (13.5%)
20	Dry eyes 136 (5.0%)	5	Sunken pulse 333 (12.2%)

### Cluster Analysis

The declining slope of total within the sum of squares moderated when the data was divided into five groups, indicating that it was an acceptable number of groups for the analysis of breast cancer patient records (**Supplementary Figure 1**).

### Symptoms and Signs of PF and SF in Each Cluster

The minimum KPI for this study of breast cancer patients was 0.231, and the best one was 0.252045. The frequency ranking differences of tongue appearances, tongue coatings, and pulses in each cluster were evaluated. According to the proportion between symptoms and signs, if the difference of an individual tongue appearance or pulse was no more than 3 among clusters, or individual coating was no more than 5 among clusters, it would be considered a PF.

The PF of breast cancer patients included insomnia, dry mouth, lack of strength, dizziness, loss of appetite, bitter taste of mouth, abdominal distention, headache, loose stool, nausea, slippery pulse, and rapid pulse. The number of cases and SF in each cluster subgroup are listed in **Table 2**.

**Table 2:** The PF and SF in each cluster subgroup.

Categories	PF	S1*	S2	S3	S4	S5
**AP**	2738	975	290	634	501	338
**Symptom**	Insomnia, dry mouth, lack of strength, dizziness, loss of appetite, bitter taste of mouth, abdominal distention, headache, loose stool, nausea	Profuse dreaming, lumbago, afraid of cold, backache, acid regurgitation, nocturia, cough, soreness	Profuse dreaming, nocturia, afraid of cold, absence of thirst, acid regurgitation	Profuse dreaming, lumbago, absence of thirst, soreness, backache, acid regurgitation, abdominal pain, cough	Lumbago, backache, soreness, profuse dreaming, dry eyes, afraid of cold, cough	Absence of thirst, cough, lumbago, backache, afraid of cold
**Pulse**	Slippery pulse, rapid pulse	Sunken pulse, weak pulse, fine pulse	String-like pulse, sunken pulse, weak pulse, fine pulse	String-like pulse, fine pulse, weak pulse, moderate pulse, sunken pulse, rough pulse	String-like pulse, fine pulse, weak pulse, sunken pulse, rough pulse, floating pulse	Moderate pulse, sunken pulse, fine pulse, weak pulse, fine pulse, rough pulse
**Tongue appearance**		Pale red tongue	Red tongue, dry tongue	Pale red tongue, red tongue, teeth-marked tongue, dark red tongue, dry tongue, enlarged tongue	Pale red tongue, teeth-marked tongue, red tongue, dark red tongue	Pale red tongue, teeth-marked tongue, red tongue
**Coating**		Slimy coating, thin coating	Slimy coating, thin coating, white coating, thick coating	Thin coating	White coating, slimy coating, thin coating	White coating, thick coating, slimy coating

*SX, Secondary features in cluster X; PF, Primary features; SF, Secondary features; AP, Amount of people.

### TCM Patterns of Combinations With Various PF and SF

**Figure 3:**
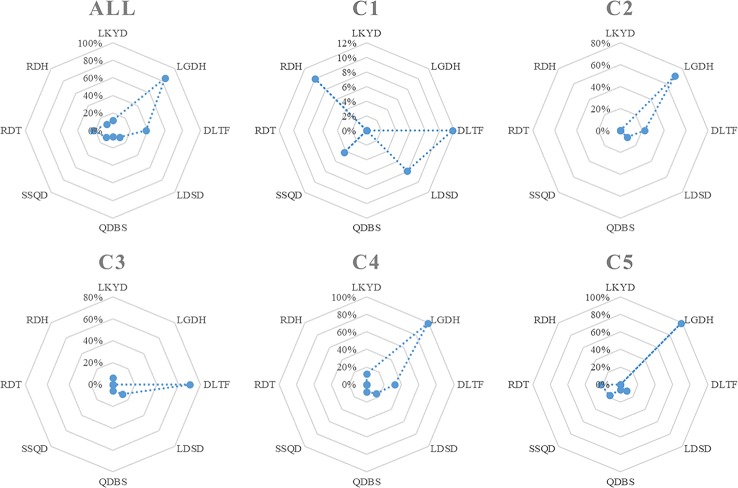
The TCM pattern distribution in each cluster subgroup. LKYD, Liver-kidney yin deficiency; LGDH, Liver-gallbladder dampness-heat; DLTF, Depressed liver qi transforming into fire; LDSD, Liver depression and spleen deficiency; QDBS, Qi deficiency with blood stasis; SSQD, Spleen-stomach qi deficiency; RDT, Retained dampness-toxin; RDH, Retained dampness-heat.

There were 87 combinations of PF and SF. The analysis of these feature combinations is demonstrated in **Supplementary Table 1**. Liver-gallbladder dampness-heat (LGDH) was the TCM pattern identified as PF. The main TCM pattern and its percentage in each cluster subgroup are demonstrated in **Table 3** and **Figure 3**. LGDH was the main TCM pattern (43%) among all feature combinations, followed by the patterns of depressed liver qi transforming into fire (DLTF) (20%) and retained dampness-toxin (RDT) (12%).

**Table 3:** Average frequency and percentage of the main TCM patterns in each cluster subgroup.

ALL	AF	%	C1*	AF	%	C2	AF	%
**LGDH**	85%	43%	DLTF	12%	35%	LGDH	70%	70%
**DLTF**	38%	20%	RDH	10%	30%	DLTF	22%	22%
**RDT**	22%	12%	LDSD	8%	23%	LDSD	9%	9%
**LKYD**	11%	6%	SSQD	4%	13%			
**LDSD**	11%	6%						
**SSQD**	11%	5%						
**RDH**	10%	5%						
**QDBS**	7%	4%						
**C3**	**AF**	**%**	**C4**	**AF**	**%**	**C5**	**AF**	**%**
**DLTF**	71%	74%	LGDH	99%	59%	LGDH	99%	64%
**LDSD**	13%	13%	DLTF	33%	19%	RDT	22%	15%
**LKYD**	6%	6%	LDSD	15%	9%	SSQD	17%	11%
**QDBS**	6%	6%	LKYD	12%	7%	LDSD	10%	7%
			QDBS	8%	5%	QDBS	6%	4%

*CX, Cluster X; TCM, Traditional Chinese medicine; AF, Average frequency; LGDH, Liver-gallbladder dampness-heat; DLTF, Depressed liver qi transforming into fire; RDT, Retained dampness-toxin; LKYD, Liver-kidney yin deficiency; LDSD, Liver depression and spleen deficiency; SSQD, Spleen-stomach qi deficiency; RDH, Retained dampness-heat; QDBS, Qi deficiency with blood stasis.

In cluster 1, DLTF was the main TCM pattern (35%), followed by retained dampness-heat (RDH) (30%), and liver depression and spleen deficiency (LDSD) (23%). LGDH was the main TCM pattern (70%) in cluster 2, followed by DLTF (22%). DLTF was the main TCM pattern (74%) in cluster 3, followed by LDSD (13%). In cluster 4, LGDH was still the main TCM pattern (59%), followed by DLTF (19%), and LDSD (9%). LGDH accounted for the main TCM pattern (64%) in cluster 5, followed by RDT (15%), and spleen-stomach qi deficiency (SSQD) (11%). For detailed definition of each pattern from WHO (World Health Organization. Regional Office for the Western, 2007), please refer to **Supplementary Table 2**.

### The Top 10 of Chinese Herbal Prescriptions in Breast Cancer Patients

As shown in **Tables 4** and **5**, the top 10 of Chinese herbal prescriptions in breast cancer patients included those that could clear heat, drain dampness and detoxify (29%), harmonize the liver and spleen (19%), tonify qi (18%), nourish the heart to tranquilize (15%), activate blood and resolve stasis (12%), tonify yin (4%), clear heat and resolve phlegm (2%), and offensive purgative (1%). The components of each formula were summarized in **Supplementary Table 3**.

**Table 4:** The top 10 of Chinese herbal prescription including single herbs and formulae in breast cancer patients in Taiwan.

Herbal prescription	Total consumption (g)*	Therapeutic Effect
***Single herb***		
*Hedyotis diffusa* Willd.	553153.5	Clear heat, drain dampness and detoxify
*Scutellaria barbata* D. Don	498634	Clear heat, drain dampness and detoxify
*Taraxacum mongolicum* Hand.-Mazz.	442880.6	Clear heat, drain dampness and detoxify
*Spatholobus suberectus* Dunn	277550.5	Activate blood and resolve stasis
*Zizyphus jujuba* Mill var. *spinosa*	236498.7	Nourish the yin to tranquilize
*Salvia miltiorrhiza* Bge.	220686.4	Activate blood and resolve stasis
*Astragalus membranaceus* (Fisch.) Bunge	209072.8	Tonify qi
*Polygonum multiflorum* Thunb.	154919.8	Nourish the heart to tranquilize
*Fritillaria thunbergii* Miq.	148233	Clear heat and resolve phlegm
*Rheum palmatum* L.	66898.8	Offensive purgative
		
***Formula***		
Jia-Wei-Xiao-Yao-San	1604612.1	Harmonize the liver and spleen
San-Zhong-Kui-Jian-Tang	523792.8	Clear heat, drain dampness and detoxify
Xue-Fu-Zhu-Yu-Tang	517921.8	Activate blood and resolve stasis
Xiang-Sha-Liu-Jun-Zi-Tang	517717.2	Tonify qi
Gui-Pi-Tang	464660	Nourish the heart to tranquilize
Bu-Zhong-Yi-Qi-Tang	403355.6	Tonify qi
Suan-Zao-Ren-Tang	402041.9	Nourish the heart to tranquilize
Zhen-Ren-Huo-Ming-Yin	388555.2	Clear heat, drain dampness and detoxify
Zhi-Bai-Di-Huang-Wan	367875	Tonify yin
Sheng-Mai-Yin	336522.3	Tonify qi

*The total consumption of the herb is the number of person-days multiplied by average daily dose.

**Table 5:** The therapeutic effects of the commonly used Chinese herbs in breast cancer patients in Taiwan.

Therapeutic effect	Total consumption (g)*	Percentage
Clear heat, drain dampness and detoxify	2407016.1	29%
Harmonize the liver and spleen	1604612.1	19%
Tonify qi	1466667.9	18%
Nourish the heart to tranquilize	1258120.4	15%
Activate blood and resolve stasis	1016158.7	12%
Tonify yin	367875	4%
Clear heat and resolve phlegm	148233	2%
Offensive purgative	66898.8	1%

*The total consumption of the herb is the number of person-days multiplied by average daily dose.

## Discussion

TCM combined with western medical treatment is widely used among breast cancer patients. Previous studies have revealed lower 5-year recurrence and metastasis rate, and decreased incidence of chronic hepatitis while receiving radiotherapy and/or chemotherapy, in breast cancer patients with the combination use of TCM (Liu et al., 2008; Huang et al., 2017). Some Chinese medicinal herbs have demonstrated effects in controlling the progression, increasing the susceptibility to radiotherapy and chemotherapy, elevating immunity, and decreasing the toxicities or side effects of cancer therapies (Yin et al., 2013). Based on the potential therapeutic effects of TCM, we explored the relationships between clinical features and TCM patterns in breast cancer patients *via* the applications of machine learning techniques. TCM clinical records were gathered in this study for text analysis.

Text analysis is a subfield of natural language processing (NLP). In the past, the lack of a widely adopted and consistently implemented medical terminology limited the use of machine-learning in medical research, especially in the field of TCM. In this study, we used the DeepMedic software to analyze unstructured electronic TCM clinical records. The software standardized and integrated key TCM terminology *via* the application of an NLP system and neural network. A total of 2,738 breast cancer records were standardized and divided into 5 subgroups *via* cluster analysis according to the frequency of clinical features reported in each case. Since patterns were not directly observable, the TCM patterns were differentiated *via* DeepMedic software by analyzing the PF and SF in each cluster subgroup.

### The TCM Patterns in Breast Cancer Patients

As shown in **Table 3** and **Figure 3**, LGDH was the main TCM pattern (43%) identified in breast cancer patients, which was compatible with the analysis of PF. According to the TCM patterns including LGDH, DLTF, and RDH, the liver is the main disease location of breast cancer, while dampness and heat were the main pathological mechanisms. According to TCM theory, the liver is related to the nerve-endocrine-immune network, it is responsible for the regulation of emotion, the promotion of digestion and absorption, and the maintenance of qi and blood circulation *via* the nerves and endocrine (Liu et al., 2017). In TCM theory, “fire” is the advanced status of “heat” in severity, while “toxin” indicates faster transmission of heat and worsening condition. Since heat and fire will damage the yin, and the depressed liver qi will impair the function of the spleen, some patients exhibit both yin and spleen qi deficiencies. Qi deficiency with blood stasis (QDBS) was also one of the SF identified in breast cancer patients, since qi deficiency will result in stagnated blood circulation. As exhibited in **Table 3** and **Figure 3**, the frequency of the LGDH and DLTF patterns had great impact on these cluster subgroups. The presence of some minor TCM patterns also helped to distinguish these five subgroups.

Cluster 1
The percentage the DLTF pattern (35%) was similar to that of the RDH pattern (30%). Additionally, the percentage of the LDSD (23%) and SSQD (13%) patterns were higher than those of other cluster subgroups. This indicates that there was no dominant TCM pattern in cluster 1.
Cluster 2
The percentages and frequencies of the TCM patterns in cluster 2 were similar to the overall cases. Compared with other patterns, the LGDH pattern was higher (70%). The secondary pattern in cluster 2 was DLTF.
Cluster 3
The percentage of the DLTF pattern was higher (74%) than in other subgroups. Patterns related with heat were the leading patterns in cluster 3. Unlike other clusters, there was no pattern related with dampness in cluster 3. Due to depressed liver qi and heat, some patients demonstrated patterns of spleen qi deficiency (13%), blood stasis (6%), and liver kidney yin deficiency (LKYD) (6%).
Cluster 4
LGDH was the dominant pattern in cluster 4, according to its high percentage (59%) and frequency (99%), followed by the DLTF pattern. Some patients demonstrated the LDSD and QDBS patterns, similar to cluster 3.
Cluster 5
LGDH was the primary pattern identified in cluster 5, with a high percentage (64%) and frequency (99%). RDT was the secondary pattern (22%) in cluster 5. Based on TCM theory, this indicates that more than one fifth of patients have a higher degree of severity and the disease develops at a faster rate.
Overall, LGDH was the leading pattern in clusters 2, 4, and 5. Meanwhile, DLTF was the secondary pattern in clusters 2 and 4, but the leading pattern in cluster 3. RDT was the secondary pattern in cluster 5. As for the patterns of spleen stomach deficiency and of blood stasis, higher frequencies were noted in clusters 4 and 5. Of note, there was no leading pattern identified in cluster 1, indicating no clear TCM pattern based on the analysis. More clinical data and information regarding clinical features are required in the TCM pattern analysis of this cluster subgroup. Clinical TCM practitioners need to exercise vigilance when treating such patients as those included in cluster 1, as these patients demonstrate no clear direction for Chinese medical treatment. As shown in **Supplementary Table 1**, the cluster analysis revealed that LGDH was the primary pattern exhibited by breast cancer patients in Taiwan.

### Associations Between TCM Patterns and Herbal Medications

According to the DeepMedic software analysis, the liver was the main viscera associated with breast cancer patients, followed by gallbladder, spleen, stomach, and kidney. Dampness, heat, and qi stagnation were the major etiologies associated with breast cancer, followed by yin deficiency, qi deficiency, and blood stasis. As shown in **Table 5**, the main therapeutic goal of TCM practitioners in Taiwan for treatment of breast cancer patients was to clear heat, drain dampness, and detoxify, consistent with the patterns of LGDH, DLTF, RDT, and RDH. This result corresponds with a previous study by Zhang et al, which reported that heat-clearing and detoxifying herbs are commonly prescribed in TCM formulas for the treatment of cancer (Zhang et al., 2017). The therapeutic principle of harmonizing the liver and spleen is consistent with LDSD. The therapeutic principle of tonifying qi is consistent with the patterns of LDSD, SSQD, and QDBS. The principle of activating blood and resolving stasis is consistent with the pattern of QDBS. The principle of tonifying yin is consistent with the pattern of LKYD. The pattern of clearing heat and resolving phlegm is consistent with the patterns of RDT and RDH. Collectively, the therapeutic principles of these Chinese herbal medications are suitable with the needs of breast cancer patients based on their TCM patterns.

### Limitations

Bias in the TCM pattern differentiation indeed exists, as it is difficult to adjust the weight based on the frequency of each clinical feature in the DeepMedic software. Moreover, selective bias may be present due to the retrieved clinical cases in this study being from a single medical center. Owing to a limited number of clinical cases, it is difficult to elucidate the TCM patterns in the different stages of breast cancer. Further study is necessary to evaluate whether different TCM patterns are related to the progression of tumor growth or to the side effects of different therapeutic modalities.

## Conclusion

This is the first study to apply a machine-learning model to standardize EMR terminology and analyze TCM patterns in breast cancer patients. With the application of neural network and cluster analyses, five primary TCM patterns were identified based on the clinical symptoms and signs reported in breast cancer patients. The therapeutic principles and prescriptions by TCM clinical practitioners focus on treating dampness, heat, and qi stagnation as the major pathologies in patients with breast cancer. In conclusion, machine learning technology could assist TCM practitioners to comprehensively differentiate patterns and identify effective Chinese herbal medicine treatments in clinical practice.

## Data Availability Statement

The datasets generated for this study are available on request to the corresponding authors.

## Ethics Statement

This study was approved by the Research Ethics Committee of China Medical University and Hospital, Taichung, Taiwan (CMUH107-REC2-023). All of the datasets analyzed were decoded so that the review board waived the requirement to sign informed consent from patients.

## Author Contributions

W-TH and H-HH equally wrote the draft and interpreted the data. W-TH, Y-WK, S-CO, H-HH, Y-CL, and Z-RY collected and assembled the data. W-TH and Y-WK analyzed the data. JL and MC provided methodological support and rectified all of analyzed data. B-CS and S-TH designed and conceived the study, and edited the manuscript. All of the authors approved the final manuscript.

## Funding

This work was supported and funded by the Ministry of Science and Technology of Taiwan (MOST 108-2320-B-039-022), Health and Welfare Surcharge of Tobacco Products, China Medical University Hospital Cancer Research Center of Excellence (MOHW108-TDU-B-212-124024), China Medical University Hospital (DMR-108-007, DMR-108-009, DMR-108-044 and CRS-108-001), An-Nan Hospital, China Medical University (ANHRF-108-06 and ANHRF-108-08) and the Chinese Medicine Research Center, China Medical University, under the Higher Education Sprout Project, Ministry of Education (CMRC-CHM-1) in Taiwan.

## Conflict of Interest

The authors declare that the research was conducted in the absence of any commercial or financial relationships that could be construed as a potential conflict of interest.

## Abbreviations

AF, Average frequency; ANN, Artificial neural networks; AP, Amount of people; CAM, Complementary and Alternative Medicine; CMUH, Chinese Medical University Hospital; DLTF, Depressed liver qi transforming into fire; EMR, Electronic medical records; ICD-9-CM, International Classification of Diseases, Ninth Revision, Clinical Modification; KPI, Key Performance Indicators; LDSD, Liver depression and spleen deficiency; LGDH, Liver-gallbladder dampness-heat; LKYD, Liver-kidney yin deficiency; NLP, Natural language processing; PF, Primary features; QDBS, Qi deficiency with blood stasis; RDH, Retained dampness-heat; RDT, Retained dampness-toxin; SF, Secondary features; SSQD, Spleen-stomach qi deficiency; TCM, Traditional Chinese medicine; TF-IDF, Term-Frequency-Inverse Document Frequency.

## References

[B1] Balneaves L. G. Bottorff J. L. Hislop T. G. Herbert C. (2006). Levels of commitment: exploring complementary therapy use by women with breast cancer. J. Altern. Complement Med. 12 (5), 459–466. 10.1089/acm.2006.12.459 16813510

[B2] Boon H. S. Olatunde F. Zick S. M. (2007). Trends in complementary/alternative medicine use by breast cancer survivors: comparing survey data from 1998 and 2005. BMC Womens Health 7 (4). 10.1186/1472-6874-7-4 PMC1851951 17397542

[B3] Chen Z. Gu K. Zheng Y. Zheng W. Lu W. Shu X. O. (2008). The use of complementary and alternative medicine among Chinese women with breast cancer. J. Altern. Complement Med. 14 (8), 1049–1055. 10.1089/acm.2008.0039 18928393 PMC6461147

[B4] Chung V. C. Wu X. Lu P. Hui E. P. Zhang Y. Zhang A. L. (2016). Chinese Herbal Medicine for Symptom Management in Cancer Palliative Care: Systematic Review And Meta-analysis. Med. (Baltimore) 95 (7), e2793. 10.1097/MD.0000000000002793 PMC4998628 26886628

[B5] Crocetti E. Crotti N. Feltrin A. Ponton P. Geddes M. Buiatti E. (1998). The use of complementary therapies by breast cancer patients attending conventional treatment. Eur. J. Cancer 34 (3), 324–328. 10.1016/s0959-8049(97)10043-0 9640216

[B6] Dodd M. Janson S. Facione N. Faucett J. Froelicher E. S. Humphreys J. (2001). Advancing the science of symptom management. J. Adv. Nurs. 33 (5), 668–676. 10.1046/j.1365-2648.2001.01697.x 11298204

[B7] Huang K. C. Yen H. R. Chiang J. H. Su Y. C. Sun M. F. Chang H. H. (2017). Chinese Herbal Medicine as an Adjunctive Therapy Ameliorated the Incidence of Chronic Hepatitis in Patients with Breast Cancer: A Nationwide Population-Based Cohort Study. Evid. Based Complement Alternat. Med. 2017, 1052976. 10.1155/2017/1052976 29234362 PMC5682887

[B8] Lin Y. C. Huang W. T. Ou S. C. Hung H. H. Cheng W. Z. Lin S. S. (2019). Neural network analysis of Chinese herbal medicine prescriptions for patients with colorectal cancer. Complement Ther. Med. 42, 279–285. 10.1016/j.ctim.2018.12.001 30670255

[B9] Ling S. Xu J. W. (2013). Model organisms and traditional chinese medicine syndrome models. Evid. Based Complement Alternat. Med. 2013, 761987. 10.1155/2013/761987 24381636 PMC3870116

[B10] Liu S. Zhao J. Liu J. Sun Z. -P. Hua Y. -Q. Lu D. -M. (2008). Effects of Ru'ai Shuhou Recipe on 5-year recurrence rate after mastectomy in breast cancer. J. Chin. Integr. Med. 6 (10), 1000–1004. 10.3736/jcim20081003 18847532

[B11] Liu Z. W. Shu J. Tu J. Y. Zhang C. H. Hong J. (2017). Liver in the Chinese and Western Medicine. Integr. Med. Int. 4 (1-2), 39–45. 10.1159/000466694

[B12] Wang Y. Yu Z. Jiang Y. Liu Y. Chen L. Liu Y. (2012). A framework and its empirical study of automatic diagnosis of traditional Chinese medicine utilizing raw free-text clinical records. J. BioMed. Inform 45 (2), 210–223. 10.1016/j.jbi.2011.10.010 22101128

[B13] World Health Organization. Regional Office for the Western, P (2007). WHO international standard terminologies on traditional medicine in the Western Pacific Region: (Manila : WHO Regional Office for the Western Pacific).

[B14] Yin S. Y. Wei W. C. Jian F. Y. Yang N. S. Therapeutic Applications of Herbal Medicines for Cancer Patients (2013). Evid. Based Complement. Alternat. Med. 2013, 302426. 10.1155/2013/302426 PMC3727181 23956768

[B15] Zhang Y. Liang Y. He C. (2017). Anticancer activities and mechanisms of heat-clearing and detoxicating traditional Chinese herbal medicine. Chin. Med. 12 (20). 10.1186/s13020-017-0140-2 PMC5506596 28702078

[B16] Zhang Z. Beck M. W. Winkler D. A. Huang B. Sibanda W. Goyal H. (2018). Opening the black box of neural networks: methods for interpreting neural network models in clinical applications. Ann. Transl. Med. 6 (11), 216. 10.21037/atm.2018.05.32 30023379 PMC6035992

