# Supplementary material for: Application of Neural Network and Cluster Analyses to Differentiate TCM Patterns in Patients With Breast Cancer
Source: Front Pharmacol. 2020 May 8;11:670. doi: 10.3389/fphar.2020.00670 (PMC7227602; doi:10.3389/fphar.2020.00670)
Supplement: Supplementary file 1 [file Table_1.docx]

| Supplementary Table 1 The patterns of 87 combinations of primary features and secondary features in each cluster | | | | |
| --- | --- | --- | --- | --- |
| P + Sx_n | **Combinations** | **Increased**  **features** | **Frequency** | **Patterns** |
| P | Insomnia,Dry mouth,Lack of strength,Dizziness,Loss of appetite,Bitter taste of mouth,Abdominal distention,Headache,Loose stool,Nausea,Slippery pulse,Rapid pulse |  |  | Liver-gallbladder dampness heat |
| P+S1_01 | Insomnia,Dry mouth,Lack of strength,Dizziness,Loss of appetite,Bitter taste of mouth,Abdominal distention,Headache,Loose stool,Nausea,Slippery pulse,Rapid pulse,Profuse dreaming | Profuse dreaming | 12% | Depressed liver qi transforming into fire |
| P+S1_02 | Insomnia,Dry mouth,Lack of strength,Dizziness,Loss of appetite,Bitter taste of mouth,Abdominal distention,Headache,Loose stool,Nausea,Slippery pulse,Rapid pulse,Profuse dreaming,Sunken pulse | Sunken pulse | 10% | Liver depression and spleen deficiency |
| P+S1_03 | Insomnia,Dry mouth,Lack of strength,Dizziness,Loss of appetite,Bitter taste of mouth,Abdominal distention,Headache,Loose stool,Nausea,Slippery pulse,Rapid pulse,Profuse dreaming,Sunken pulse,Slimy coating | Slimy coating | 10% | Retained dampness heat |
| P+S1_04 | Insomnia,Dry mouth,Lack of strength,Dizziness,Loss of appetite,Bitter taste of mouth,Abdominal distention,Headache,Loose stool,Nausea,Slippery pulse,Rapid pulse,Profuse dreaming,Sunken pulse,Slimy coating,Pale red tongue | Pale red tongue | 10% | Liver depression and spleen deficiency |
| P+S1_05 | Insomnia,Dry mouth,Lack of strength,Dizziness,Loss of appetite,Bitter taste of mouth,Abdominal distention,Headache,Loose stool,Nausea,Slippery pulse,Rapid pulse,Profuse dreaming,Sunken pulse,Slimy coating,Pale red tongue,Weak pulse | Weak pulse | 9% | Liver depression and spleen deficiency |
| P+S1_06 | Insomnia,Dry mouth,Lack of strength,Dizziness,Loss of appetite,Bitter taste of mouth,Abdominal distention,Headache,Loose stool,Nausea,Slippery pulse,Rapid pulse,Profuse dreaming,Sunken pulse,Slimy coating,Pale red tongue,Weak pulse,Lumbago | Lumbago | 8% | Liver depression and spleen deficiency |
| P+S1_07 | Insomnia,Dry mouth,Lack of strength,Dizziness,Loss of appetite,Bitter taste of mouth,Abdominal distention,Headache,Loose stool,Nausea,Slippery pulse,Rapid pulse,Profuse dreaming,Sunken pulse,Slimy coating,Pale red tongue,Weak pulse,Lumbago,Fine pulse | Fine pulse | 8% | Liver depression and spleen deficiency |
| P+S1_08 | Insomnia,Dry mouth,Lack of strength,Dizziness,Loss of appetite,Bitter taste of mouth,Abdominal distention,Headache,Loose stool,Nausea,Slippery pulse,Rapid pulse,Profuse dreaming,Sunken pulse,Slimy coating,Pale red tongue,Weak pulse,Lumbago,Fine pulse,Afraid of cold | Afraid of cold | 8% | Liver depression and spleen deficiency |
| P+S1_09 | Insomnia,Dry mouth,Lack of strength,Dizziness,Loss of appetite,Bitter taste of mouth,Abdominal distention,Headache,Loose stool,Nausea,Slippery pulse,Rapid pulse,Profuse dreaming,Sunken pulse,Slimy coating,Pale red tongue,Weak pulse,Lumbago,Fine pulse,Afraid of cold,Backache | Backache | 7% | Liver depression and spleen deficiency |
| P+S1_10 | Insomnia,Dry mouth,Lack of strength,Dizziness,Loss of appetite,Bitter taste of mouth,Abdominal distention,Headache,Loose stool,Nausea,Slippery pulse,Rapid pulse,Profuse dreaming,Sunken pulse,Slimy coating,Pale red tongue,Weak pulse,Lumbago,Fine pulse,Afraid of cold,Backache,Acid regurgitation | Acid regurgitation | 7% | Liver depression and spleen deficiency |
| P+S1_11 | Insomnia,Dry mouth,Lack of strength,Dizziness,Loss of appetite,Bitter taste of mouth,Abdominal distention,Headache,Loose stool,Nausea,Slippery pulse,Rapid pulse,Profuse dreaming,Sunken pulse,Slimy coating,Pale red tongue,Weak pulse,Lumbago,Fine pulse,Afraid of cold,Backache,Acid regurgitation,Nocturia | Nocturia | 7% | Liver depression and spleen deficiency |
| P+S1_12 | Insomnia,Dry mouth,Lack of strength,Dizziness,Loss of appetite,Bitter taste of mouth,Abdominal distention,Headache,Loose stool,Nausea,Slippery pulse,Rapid pulse,Profuse dreaming,Sunken pulse,Slimy coating,Pale red tongue,Weak pulse,Lumbago,Fine pulse,Afraid of cold,Backache,Acid regurgitation,Nocturia,Thin coating | Thin coating | 6% | Liver depression and spleen deficiency |
| P+S1_13 | Insomnia,Dry mouth,Lack of strength,Dizziness,Loss of appetite,Bitter taste of mouth,Abdominal distention,Headache,Loose stool,Nausea,Slippery pulse,Rapid pulse,Profuse dreaming,Sunken pulse,Slimy coating,Pale red tongue,Weak pulse,Lumbago,Fine pulse,Afraid of cold,Backache,Acid regurgitation,Nocturia,Thin coating,Cough | Cough | 6% | Liver depression and spleen deficiency |
| P+S1_14 | Insomnia,Dry mouth,Lack of strength,Dizziness,Loss of appetite,Bitter taste of mouth,Abdominal distention,Headache,Loose stool,Nausea,Slippery pulse,Rapid pulse,Profuse dreaming,Sunken pulse,Slimy coating,Pale red tongue,Weak pulse,Lumbago,Fine pulse,Afraid of cold,Backache,Acid regurgitation,Nocturia,Thin coating,Cough,Soreness | Soreness | 4% | Spleen stomach qi deficiency |
| P+S2_01 | Insomnia,Dry mouth,Lack of strength,Dizziness,Loss of appetite,Bitter taste of mouth,Abdominal distention,Headache,Loose stool,Nausea,Slippery pulse,Rapid pulse,Red tongue | Red tongue | 100% | Liver-gallbladder dampness heat |
| P+S2_02 | Insomnia,Dry mouth,Lack of strength,Dizziness,Loss of appetite,Bitter taste of mouth,Abdominal distention,Headache,Loose stool,Nausea,Slippery pulse,Rapid pulse,Red tongue,Slimy coating | Slimy coating | 41% | Liver-gallbladder dampness heat |
| P+S2_03 | Insomnia,Dry mouth,Lack of strength,Dizziness,Loss of appetite,Bitter taste of mouth,Abdominal distention,Headache,Loose stool,Nausea,Slippery pulse,Rapid pulse,Red tongue,Slimy coating,Thin coating | Thin coating | 26% | Depressed liver qi transforming into fire |
| P+S2_04 | Insomnia,Dry mouth,Lack of strength,Dizziness,Loss of appetite,Bitter taste of mouth,Abdominal distention,Headache,Loose stool,Nausea,Slippery pulse,Rapid pulse,Red tongue,Slimy coating,Thin coating,String-like pulse | String-like pulse | 19% | Depressed liver qi transforming into fire |
| P+S2_05 | Insomnia,Dry mouth,Lack of strength,Dizziness,Loss of appetite,Bitter taste of mouth,Abdominal distention,Headache,Loose stool,Nausea,Slippery pulse,Rapid pulse,Red tongue,Slimy coating,Thin coating,String-like pulse,Sunken pulse | Sunken pulse | 12% | Liver depression and spleen deficiency |
| P+S2_06 | Insomnia,Dry mouth,Lack of strength,Dizziness,Loss of appetite,Bitter taste of mouth,Abdominal distention,Headache,Loose stool,Nausea,Slippery pulse,Rapid pulse,Red tongue,Slimy coating,Thin coating,String-like pulse,Sunken pulse,Profuse dreaming | Profuse dreaming | 13% | Liver depression and spleen deficiency |
| P+S2_07 | Insomnia,Dry mouth,Lack of strength,Dizziness,Loss of appetite,Bitter taste of mouth,Abdominal distention,Headache,Loose stool,Nausea,Slippery pulse,Rapid pulse,Red tongue,Slimy coating,Thin coating,String-like pulse,Sunken pulse,Profuse dreaming,White coating | White coating | 11% | Liver depression and spleen deficiency |
| P+S2_08 | Insomnia,Dry mouth,Lack of strength,Dizziness,Loss of appetite,Bitter taste of mouth,Abdominal distention,Headache,Loose stool,Nausea,Slippery pulse,Rapid pulse,Red tongue,Slimy coating,Thin coating,String-like pulse,Sunken pulse,Profuse dreaming,White coating,Nocturia | Nocturia | 10% | Liver depression and spleen deficiency |
| P+S2_09 | Insomnia,Dry mouth,Lack of strength,Dizziness,Loss of appetite,Bitter taste of mouth,Abdominal distention,Headache,Loose stool,Nausea,Slippery pulse,Rapid pulse,Red tongue,Slimy coating,Thin coating,String-like pulse,Sunken pulse,Profuse dreaming,White coating,Nocturia,Weak pulse | Weak pulse | 9% | Liver depression and spleen deficiency |
| P+S2_10 | Insomnia,Dry mouth,Lack of strength,Dizziness,Loss of appetite,Bitter taste of mouth,Abdominal distention,Headache,Loose stool,Nausea,Slippery pulse,Rapid pulse,Red tongue,Slimy coating,Thin coating,String-like pulse,Sunken pulse,Profuse dreaming,White coating,Nocturia,Weak pulse,Dry tongue | Dry tongue | 9% | Liver depression and spleen deficiency |
| P+S2_11 | Insomnia,Dry mouth,Lack of strength,Dizziness,Loss of appetite,Bitter taste of mouth,Abdominal distention,Headache,Loose stool,Nausea,Slippery pulse,Rapid pulse,Red tongue,Slimy coating,Thin coating,String-like pulse,Sunken pulse,Profuse dreaming,White coating,Nocturia,Weak pulse,Dry tongue,Afraid of cold | Afraid of cold | 8% | Liver depression and spleen deficiency |
| P+S2_12 | Insomnia,Dry mouth,Lack of strength,Dizziness,Loss of appetite,Bitter taste of mouth,Abdominal distention,Headache,Loose stool,Nausea,Slippery pulse,Rapid pulse,Red tongue,Slimy coating,Thin coating,String-like pulse,Sunken pulse,Profuse dreaming,White coating,Nocturia,Weak pulse,Dry tongue,Afraid of cold,Absence of thirst | Absence of thirst | 7% | Liver depression and spleen deficiency |
| P+S2_13 | Insomnia,Dry mouth,Lack of strength,Dizziness,Loss of appetite,Bitter taste of mouth,Abdominal distention,Headache,Loose stool,Nausea,Slippery pulse,Rapid pulse,Red tongue,Slimy coating,Thin coating,String-like pulse,Sunken pulse,Profuse dreaming,White coating,Nocturia,Weak pulse,Dry tongue,Afraid of cold,Absence of thirst,Acid regurgitation | Acid regurgitation | 6% | Liver depression and spleen deficiency |
| P+S2_14 | Insomnia,Dry mouth,Lack of strength,Dizziness,Loss of appetite,Bitter taste of mouth,Abdominal distention,Headache,Loose stool,Nausea,Slippery pulse,Rapid pulse,Red tongue,Slimy coating,Thin coating,String-like pulse,Sunken pulse,Profuse dreaming,White coating,Nocturia,Weak pulse,Dry tongue,Afraid of cold,Absence of thirst,Acid regurgitation,Fine pulse | Fine pulse | 6% | Liver depression and spleen deficiency |
| P+S2_15 | Insomnia,Dry mouth,Lack of strength,Dizziness,Loss of appetite,Bitter taste of mouth,Abdominal distention,Headache,Loose stool,Nausea,Slippery pulse,Rapid pulse,Red tongue,Slimy coating,Thin coating,String-like pulse,Sunken pulse,Profuse dreaming,White coating,Nocturia,Weak pulse,Dry tongue,Afraid of cold,Absence of thirst,Acid regurgitation,Fine pulse,Thick coating | Thick coating | 5% | Liver depression and spleen deficiency |
| P+S3_01 | Insomnia,Dry mouth,Lack of strength,Dizziness,Loss of appetite,Bitter taste of mouth,Abdominal distention,Headache,Loose stool,Nausea,Slippery pulse,Rapid pulse,Thin coating | Thin coating | 100% | Depressed liver qi transforming into fire |
| P+S3_02 | Insomnia,Dry mouth,Lack of strength,Dizziness,Loss of appetite,Bitter taste of mouth,Abdominal distention,Headache,Loose stool,Nausea,Slippery pulse,Rapid pulse,Thin coating,String-like pulse | String-like pulse | 41% | Depressed liver qi transforming into fire |
| P+S3_03 | Insomnia,Dry mouth,Lack of strength,Dizziness,Loss of appetite,Bitter taste of mouth,Abdominal distention,Headache,Loose stool,Nausea,Slippery pulse,Rapid pulse,Thin coating,String-like pulse,Pale red tongue | Pale red tongue | 35% | Liver depression and spleen deficiency |
| P+S3_04 | Insomnia,Dry mouth,Lack of strength,Dizziness,Loss of appetite,Bitter taste of mouth,Abdominal distention,Headache,Loose stool,Nausea,Slippery pulse,Rapid pulse,Thin coating,String-like pulse,Pale red tongue,Fine pulse | Fine pulse | 21% | Liver depression and spleen deficiency |
| P+S3_05 | Insomnia,Dry mouth,Lack of strength,Dizziness,Loss of appetite,Bitter taste of mouth,Abdominal distention,Headache,Loose stool,Nausea,Slippery pulse,Rapid pulse,Thin coating,String-like pulse,Pale red tongue,Fine pulse,Red tongue | Red tongue | 16% | Liver depression and spleen deficiency |
| P+S3_06 | Insomnia,Dry mouth,Lack of strength,Dizziness,Loss of appetite,Bitter taste of mouth,Abdominal distention,Headache,Loose stool,Nausea,Slippery pulse,Rapid pulse,Thin coating,String-like pulse,Pale red tongue,Fine pulse,Red tongue,Weak pulse | Weak pulse | 15% | Liver depression and spleen deficiency |
| P+S3_07 | Insomnia,Dry mouth,Lack of strength,Dizziness,Loss of appetite,Bitter taste of mouth,Abdominal distention,Headache,Loose stool,Nausea,Slippery pulse,Rapid pulse,Thin coating,String-like pulse,Pale red tongue,Fine pulse,Red tongue,Weak pulse,Moderate pulse | Moderate pulse | 15% | Liver depression and spleen deficiency |
| P+S3_08 | Insomnia,Dry mouth,Lack of strength,Dizziness,Loss of appetite,Bitter taste of mouth,Abdominal distention,Headache,Loose stool,Nausea,Slippery pulse,Rapid pulse,Thin coating,String-like pulse,Pale red tongue,Fine pulse,Red tongue,Weak pulse,Moderate pulse,Teeth-marked tongue | Teeth-marked tongue | 14% | Liver depression and spleen deficiency |
| P+S3_09 | Insomnia,Dry mouth,Lack of strength,Dizziness,Loss of appetite,Bitter taste of mouth,Abdominal distention,Headache,Loose stool,Nausea,Slippery pulse,Rapid pulse,Thin coating,String-like pulse,Pale red tongue,Fine pulse,Red tongue,Weak pulse,Moderate pulse,Teeth-marked tongue,Sunken pulse | Sunken pulse | 11% | Liver depression and spleen deficiency |
| P+S3_10 | Insomnia,Dry mouth,Lack of strength,Dizziness,Loss of appetite,Bitter taste of mouth,Abdominal distention,Headache,Loose stool,Nausea,Slippery pulse,Rapid pulse,Thin coating,String-like pulse,Pale red tongue,Fine pulse,Red tongue,Weak pulse,Moderate pulse,Teeth-marked tongue,Sunken pulse,Rough pulse | Rough pulse | 11% | Liver depression and spleen deficiency |
| P+S3_11 | Insomnia,Dry mouth,Lack of strength,Dizziness,Loss of appetite,Bitter taste of mouth,Abdominal distention,Headache,Loose stool,Nausea,Slippery pulse,Rapid pulse,Thin coating,String-like pulse,Pale red tongue,Fine pulse,Red tongue,Weak pulse,Moderate pulse,Teeth-marked tongue,Sunken pulse,Rough pulse,Profuse dreaming | Profuse dreaming | 9% | Liver depression and spleen deficiency |
| P+S3_12 | Insomnia,Dry mouth,Lack of strength,Dizziness,Loss of appetite,Bitter taste of mouth,Abdominal distention,Headache,Loose stool,Nausea,Slippery pulse,Rapid pulse,Thin coating,String-like pulse,Pale red tongue,Fine pulse,Red tongue,Weak pulse,Moderate pulse,Teeth-marked tongue,Sunken pulse,Rough pulse,Profuse dreaming,Lumbago | Lumbago | 8% | Liver depression and spleen deficiency |
| P+S3_13 | Insomnia,Dry mouth,Lack of strength,Dizziness,Loss of appetite,Bitter taste of mouth,Abdominal distention,Headache,Loose stool,Nausea,Slippery pulse,Rapid pulse,Thin coating,String-like pulse,Pale red tongue,Fine pulse,Red tongue,Weak pulse,Moderate pulse,Teeth-marked tongue,Sunken pulse,Rough pulse,Profuse dreaming,Lumbago,Absence of thirst | Absence of thirst | 9% | Liver depression and spleen deficiency |
| P+S3_14 | Insomnia,Dry mouth,Lack of strength,Dizziness,Loss of appetite,Bitter taste of mouth,Abdominal distention,Headache,Loose stool,Nausea,Slippery pulse,Rapid pulse,Thin coating,String-like pulse,Pale red tongue,Fine pulse,Red tongue,Weak pulse,Moderate pulse,Teeth-marked tongue,Sunken pulse,Rough pulse,Profuse dreaming,Lumbago,Absence of thirst,Soreness | Soreness | 7% | Liver depression and spleen deficiency |
| P+S3_15 | Insomnia,Dry mouth,Lack of strength,Dizziness,Loss of appetite,Bitter taste of mouth,Abdominal distention,Headache,Loose stool,Nausea,Slippery pulse,Rapid pulse,Thin coating,String-like pulse,Pale red tongue,Fine pulse,Red tongue,Weak pulse,Moderate pulse,Teeth-marked tongue,Sunken pulse,Rough pulse,Profuse dreaming,Lumbago,Absence of thirst,Soreness,Backache | Backache | 7% | Liver depression and spleen deficiency |
| P+S3_16 | Insomnia,Dry mouth,Lack of strength,Dizziness,Loss of appetite,Bitter taste of mouth,Abdominal distention,Headache,Loose stool,Nausea,Slippery pulse,Rapid pulse,Thin coating,String-like pulse,Pale red tongue,Fine pulse,Red tongue,Weak pulse,Moderate pulse,Teeth-marked tongue,Sunken pulse,Rough pulse,Profuse dreaming,Lumbago,Absence of thirst,Soreness,Backache,Dark red tongue | Dark red tongue | 6% | Qi deficiency with blood stasis |
| P+S3_17 | Insomnia,Dry mouth,Lack of strength,Dizziness,Loss of appetite,Bitter taste of mouth,Abdominal distention,Headache,Loose stool,Nausea,Slippery pulse,Rapid pulse,Thin coating,String-like pulse,Pale red tongue,Fine pulse,Red tongue,Weak pulse,Moderate pulse,Teeth-marked tongue,Sunken pulse,Rough pulse,Profuse dreaming,Lumbago,Absence of thirst,Soreness,Backache,Dark red tongue,Acid regurgitation | Acid regurgitation | 6% | Liver depression and spleen deficiency |
| P+S3_18 | Insomnia,Dry mouth,Lack of strength,Dizziness,Loss of appetite,Bitter taste of mouth,Abdominal distention,Headache,Loose stool,Nausea,Slippery pulse,Rapid pulse,Thin coating,String-like pulse,Pale red tongue,Fine pulse,Red tongue,Weak pulse,Moderate pulse,Teeth-marked tongue,Sunken pulse,Rough pulse,Profuse dreaming,Lumbago,Absence of thirst,Soreness,Backache,Dark red tongue,Acid regurgitation,Dry tongue | Dry tongue | 6% | Liver-kidney yin deficiency |
| P+S3_19 | Insomnia,Dry mouth,Lack of strength,Dizziness,Loss of appetite,Bitter taste of mouth,Abdominal distention,Headache,Loose stool,Nausea,Slippery pulse,Rapid pulse,Thin coating,String-like pulse,Pale red tongue,Fine pulse,Red tongue,Weak pulse,Moderate pulse,Teeth-marked tongue,Sunken pulse,Rough pulse,Profuse dreaming,Lumbago,Absence of thirst,Soreness,Backache,Dark red tongue,Acid regurgitation,Dry tongue,Abdominal pain | Abdominal pain | 6% | Liver depression and spleen deficiency |
| P+S3_20 | Insomnia,Dry mouth,Lack of strength,Dizziness,Loss of appetite,Bitter taste of mouth,Abdominal distention,Headache,Loose stool,Nausea,Slippery pulse,Rapid pulse,Thin coating,String-like pulse,Pale red tongue,Fine pulse,Red tongue,Weak pulse,Moderate pulse,Teeth-marked tongue,Sunken pulse,Rough pulse,Profuse dreaming,Lumbago,Absence of thirst,Soreness,Backache,Dark red tongue,Acid regurgitation,Dry tongue,Abdominal pain,Cough | Cough | 6% | Qi deficiency with blood stasis |
| P+S3_21 | Insomnia,Dry mouth,Lack of strength,Dizziness,Loss of appetite,Bitter taste of mouth,Abdominal distention,Headache,Loose stool,Nausea,Slippery pulse,Rapid pulse,Thin coating,String-like pulse,Pale red tongue,Fine pulse,Red tongue,Weak pulse,Moderate pulse,Teeth-marked tongue,Sunken pulse,Rough pulse,Profuse dreaming,Lumbago,Absence of thirst,Soreness,Backache,Dark red tongue,Acid regurgitation,Dry tongue,Abdominal pain,Cough,Enlarged tongue | Enlarged tongue | 5% | Qi deficiency with blood stasis |
| P+S4_01 | Insomnia,Dry mouth,Lack of strength,Dizziness,Loss of appetite,Bitter taste of mouth,Abdominal distention,Headache,Loose stool,Nausea,Slippery pulse,Rapid pulse,String-like pulse | String-like pulse | 99% | Liver-gallbladder dampness heat |
| P+S4_02 | Insomnia,Dry mouth,Lack of strength,Dizziness,Loss of appetite,Bitter taste of mouth,Abdominal distention,Headache,Loose stool,Nausea,Slippery pulse,Rapid pulse,String-like pulse,Fine pulse | Fine pulse | 36% | Liver-kidney yin deficiency |
| P+S4_03 | Insomnia,Dry mouth,Lack of strength,Dizziness,Loss of appetite,Bitter taste of mouth,Abdominal distention,Headache,Loose stool,Nausea,Slippery pulse,Rapid pulse,String-like pulse,Fine pulse,White coating | White coating | 33% | Depressed liver qi transforming into fire |
| P+S4_04 | Insomnia,Dry mouth,Lack of strength,Dizziness,Loss of appetite,Bitter taste of mouth,Abdominal distention,Headache,Loose stool,Nausea,Slippery pulse,Rapid pulse,String-like pulse,Fine pulse,White coating,Pale red tongue | Pale red tongue | 23% | Liver depression and spleen deficiency |
| P+S4_05 | Insomnia,Dry mouth,Lack of strength,Dizziness,Loss of appetite,Bitter taste of mouth,Abdominal distention,Headache,Loose stool,Nausea,Slippery pulse,Rapid pulse,String-like pulse,Fine pulse,White coating,Pale red tongue,Weak pulse | Weak pulse | 22% | Liver depression and spleen deficiency |
| P+S4_06 | Insomnia,Dry mouth,Lack of strength,Dizziness,Loss of appetite,Bitter taste of mouth,Abdominal distention,Headache,Loose stool,Nausea,Slippery pulse,Rapid pulse,String-like pulse,Fine pulse,White coating,Pale red tongue,Weak pulse,Slimy coating | Slimy coating | 17% | Liver depression and spleen deficiency |
| P+S4_07 | Insomnia,Dry mouth,Lack of strength,Dizziness,Loss of appetite,Bitter taste of mouth,Abdominal distention,Headache,Loose stool,Nausea,Slippery pulse,Rapid pulse,String-like pulse,Fine pulse,White coating,Pale red tongue,Weak pulse,Slimy coating,Sunken pulse | Sunken pulse | 14% | Liver depression and spleen deficiency |
| P+S4_08 | Insomnia,Dry mouth,Lack of strength,Dizziness,Loss of appetite,Bitter taste of mouth,Abdominal distention,Headache,Loose stool,Nausea,Slippery pulse,Rapid pulse,String-like pulse,Fine pulse,White coating,Pale red tongue,Weak pulse,Slimy coating,Sunken pulse,Lumbago | Lumbago | 14% | Liver depression and spleen deficiency |
| P+S4_09 | Insomnia,Dry mouth,Lack of strength,Dizziness,Loss of appetite,Bitter taste of mouth,Abdominal distention,Headache,Loose stool,Nausea,Slippery pulse,Rapid pulse,String-like pulse,Fine pulse,White coating,Pale red tongue,Weak pulse,Slimy coating,Sunken pulse,Lumbago,Teeth-marked tongue | Teeth-marked tongue | 12% | Liver depression and spleen deficiency |
| P+S4_10 | Insomnia,Dry mouth,Lack of strength,Dizziness,Loss of appetite,Bitter taste of mouth,Abdominal distention,Headache,Loose stool,Nausea,Slippery pulse,Rapid pulse,String-like pulse,Fine pulse,White coating,Pale red tongue,Weak pulse,Slimy coating,Sunken pulse,Lumbago,Teeth-marked tongue,Backache | Backache | 10% | Liver depression and spleen deficiency |
| P+S4_11 | Insomnia,Dry mouth,Lack of strength,Dizziness,Loss of appetite,Bitter taste of mouth,Abdominal distention,Headache,Loose stool,Nausea,Slippery pulse,Rapid pulse,String-like pulse,Fine pulse,White coating,Pale red tongue,Weak pulse,Slimy coating,Sunken pulse,Lumbago,Teeth-marked tongue,Backache,Rough pulse | Rough pulse | 10% | Qi deficiency with blood stasis |
| P+S4_12 | Insomnia,Dry mouth,Lack of strength,Dizziness,Loss of appetite,Bitter taste of mouth,Abdominal distention,Headache,Loose stool,Nausea,Slippery pulse,Rapid pulse,String-like pulse,Fine pulse,White coating,Pale red tongue,Weak pulse,Slimy coating,Sunken pulse,Lumbago,Teeth-marked tongue,Backache,Rough pulse,Red tongue | Red tongue | 9% | Liver depression and spleen deficiency |
| P+S4_13 | Insomnia,Dry mouth,Lack of strength,Dizziness,Loss of appetite,Bitter taste of mouth,Abdominal distention,Headache,Loose stool,Nausea,Slippery pulse,Rapid pulse,String-like pulse,Fine pulse,White coating,Pale red tongue,Weak pulse,Slimy coating,Sunken pulse,Lumbago,Teeth-marked tongue,Backache,Rough pulse,Red tongue,Dark red tongue | Dark red tongue | 8% | Qi deficiency with blood stasis |
| P+S4_14 | Insomnia,Dry mouth,Lack of strength,Dizziness,Loss of appetite,Bitter taste of mouth,Abdominal distention,Headache,Loose stool,Nausea,Slippery pulse,Rapid pulse,String-like pulse,Fine pulse,White coating,Pale red tongue,Weak pulse,Slimy coating,Sunken pulse,Lumbago,Teeth-marked tongue,Backache,Rough pulse,Red tongue,Dark red tongue,Soreness | Soreness | 8% | Qi deficiency with blood stasis |
| P+S4_15 | Insomnia,Dry mouth,Lack of strength,Dizziness,Loss of appetite,Bitter taste of mouth,Abdominal distention,Headache,Loose stool,Nausea,Slippery pulse,Rapid pulse,String-like pulse,Fine pulse,White coating,Pale red tongue,Weak pulse,Slimy coating,Sunken pulse,Lumbago,Teeth-marked tongue,Backache,Rough pulse,Red tongue,Dark red tongue,Soreness,Profuse dreaming | Profuse dreaming | 8% | Qi deficiency with blood stasis |
| P+S4_16 | Insomnia,Dry mouth,Lack of strength,Dizziness,Loss of appetite,Bitter taste of mouth,Abdominal distention,Headache,Loose stool,Nausea,Slippery pulse,Rapid pulse,String-like pulse,Fine pulse,White coating,Pale red tongue,Weak pulse,Slimy coating,Sunken pulse,Lumbago,Teeth-marked tongue,Backache,Rough pulse,Red tongue,Dark red tongue,Soreness,Profuse dreaming,Dry eyes | Dry eyes | 8% | Liver-kidney yin deficiency |
| P+S4_17 | Insomnia,Dry mouth,Lack of strength,Dizziness,Loss of appetite,Bitter taste of mouth,Abdominal distention,Headache,Loose stool,Nausea,Slippery pulse,Rapid pulse,String-like pulse,Fine pulse,White coating,Pale red tongue,Weak pulse,Slimy coating,Sunken pulse,Lumbago,Teeth-marked tongue,Backache,Rough pulse,Red tongue,Dark red tongue,Soreness,Profuse dreaming,Dry eyes,Afraid of cold | Afraid of cold | 8% | Liver-kidney yin deficiency |
| P+S4_18 | Insomnia,Dry mouth,Lack of strength,Dizziness,Loss of appetite,Bitter taste of mouth,Abdominal distention,Headache,Loose stool,Nausea,Slippery pulse,Rapid pulse,String-like pulse,Fine pulse,White coating,Pale red tongue,Weak pulse,Slimy coating,Sunken pulse,Lumbago,Teeth-marked tongue,Backache,Rough pulse,Red tongue,Dark red tongue,Soreness,Profuse dreaming,Dry eyes,Afraid of cold,Thin coating | Thin coating | 7% | Liver-kidney yin deficiency |
| P+S4_19 | Insomnia,Dry mouth,Lack of strength,Dizziness,Loss of appetite,Bitter taste of mouth,Abdominal distention,Headache,Loose stool,Nausea,Slippery pulse,Rapid pulse,String-like pulse,Fine pulse,White coating,Pale red tongue,Weak pulse,Slimy coating,Sunken pulse,Lumbago,Teeth-marked tongue,Backache,Rough pulse,Red tongue,Dark red tongue,Soreness,Profuse dreaming,Dry eyes,Afraid of cold,Thin coating,Cough | Cough | 7% | Liver-kidney yin deficiency |
| P+S4_20 | Insomnia,Dry mouth,Lack of strength,Dizziness,Loss of appetite,Bitter taste of mouth,Abdominal distention,Headache,Loose stool,Nausea,Slippery pulse,Rapid pulse,String-like pulse,Fine pulse,White coating,Pale red tongue,Weak pulse,Slimy coating,Sunken pulse,Lumbago,Teeth-marked tongue,Backache,Rough pulse,Red tongue,Dark red tongue,Soreness,Profuse dreaming,Dry eyes,Afraid of cold,Thin coating,Cough,Floating pulse | Floating pulse | 6% | Liver-kidney yin deficiency |
| P+S5_01 | Insomnia,Dry mouth,Lack of strength,Dizziness,Loss of appetite,Bitter taste of mouth,Abdominal distention,Headache,Loose stool,Nausea,Slippery pulse,Rapid pulse,White coating | White coating | 99% | Liver-gallbladder dampness heat |
| P+S5_02 | Insomnia,Dry mouth,Lack of strength,Dizziness,Loss of appetite,Bitter taste of mouth,Abdominal distention,Headache,Loose stool,Nausea,Slippery pulse,Rapid pulse,White coating,Thick coating | Thick coating | 50% | Retained dampness toxin |
| P+S5_03 | Insomnia,Dry mouth,Lack of strength,Dizziness,Loss of appetite,Bitter taste of mouth,Abdominal distention,Headache,Loose stool,Nausea,Slippery pulse,Rapid pulse,White coating,Thick coating,Pale red tongue | Pale red tongue | 25% | Retained dampness toxin |
| P+S5_04 | Insomnia,Dry mouth,Lack of strength,Dizziness,Loss of appetite,Bitter taste of mouth,Abdominal distention,Headache,Loose stool,Nausea,Slippery pulse,Rapid pulse,White coating,Thick coating,Pale red tongue,Moderate pulse | Moderate pulse | 20% | Retained dampness toxin |
| P+S5_05 | Insomnia,Dry mouth,Lack of strength,Dizziness,Loss of appetite,Bitter taste of mouth,Abdominal distention,Headache,Loose stool,Nausea,Slippery pulse,Rapid pulse,White coating,Thick coating,Pale red tongue,Moderate pulse,Slimy coating | Slimy coating | 17% | Spleen stomach qi deficiency |
| P+S5_06 | Insomnia,Dry mouth,Lack of strength,Dizziness,Loss of appetite,Bitter taste of mouth,Abdominal distention,Headache,Loose stool,Nausea,Slippery pulse,Rapid pulse,White coating,Thick coating,Pale red tongue,Moderate pulse,Slimy coating,Sunken pulse | Sunken pulse | 17% | Retained dampness toxin |
| P+S5_07 | Insomnia,Dry mouth,Lack of strength,Dizziness,Loss of appetite,Bitter taste of mouth,Abdominal distention,Headache,Loose stool,Nausea,Slippery pulse,Rapid pulse,White coating,Thick coating,Pale red tongue,Moderate pulse,Slimy coating,Sunken pulse,String-like pulse | String-like pulse | 16% | Retained dampness toxin |
| P+S5_08 | Insomnia,Dry mouth,Lack of strength,Dizziness,Loss of appetite,Bitter taste of mouth,Abdominal distention,Headache,Loose stool,Nausea,Slippery pulse,Rapid pulse,White coating,Thick coating,Pale red tongue,Moderate pulse,Slimy coating,Sunken pulse,String-like pulse,Weak pulse | Weak pulse | 13% | Liver depression and spleen deficiency |
| P+S5_09 | Insomnia,Dry mouth,Lack of strength,Dizziness,Loss of appetite,Bitter taste of mouth,Abdominal distention,Headache,Loose stool,Nausea,Slippery pulse,Rapid pulse,White coating,Thick coating,Pale red tongue,Moderate pulse,Slimy coating,Sunken pulse,String-like pulse,Weak pulse,Teeth-marked tongue | Teeth-marked tongue | 12% | Liver depression and spleen deficiency |
| P+S5_10 | Insomnia,Dry mouth,Lack of strength,Dizziness,Loss of appetite,Bitter taste of mouth,Abdominal distention,Headache,Loose stool,Nausea,Slippery pulse,Rapid pulse,White coating,Thick coating,Pale red tongue,Moderate pulse,Slimy coating,Sunken pulse,String-like pulse,Weak pulse,Teeth-marked tongue,Fine pulse | Fine pulse | 12% | Liver depression and spleen deficiency |
| P+S5_11 | Insomnia,Dry mouth,Lack of strength,Dizziness,Loss of appetite,Bitter taste of mouth,Abdominal distention,Headache,Loose stool,Nausea,Slippery pulse,Rapid pulse,White coating,Thick coating,Pale red tongue,Moderate pulse,Slimy coating,Sunken pulse,String-like pulse,Weak pulse,Teeth-marked tongue,Fine pulse,Rough pulse | Rough pulse | 11% | Liver depression and spleen deficiency |
| P+S5_12 | Insomnia,Dry mouth,Lack of strength,Dizziness,Loss of appetite,Bitter taste of mouth,Abdominal distention,Headache,Loose stool,Nausea,Slippery pulse,Rapid pulse,White coating,Thick coating,Pale red tongue,Moderate pulse,Slimy coating,Sunken pulse,String-like pulse,Weak pulse,Teeth-marked tongue,Fine pulse,Rough pulse,Absence of thirst | Absence of thirst | 10% | Liver depression and spleen deficiency |
| P+S5_13 | Insomnia,Dry mouth,Lack of strength,Dizziness,Loss of appetite,Bitter taste of mouth,Abdominal distention,Headache,Loose stool,Nausea,Slippery pulse,Rapid pulse,White coating,Thick coating,Pale red tongue,Moderate pulse,Slimy coating,Sunken pulse,String-like pulse,Weak pulse,Teeth-marked tongue,Fine pulse,Rough pulse,Absence of thirst,Cough | Cough | 8% | Liver depression and spleen deficiency |
| P+S5_14 | Insomnia,Dry mouth,Lack of strength,Dizziness,Loss of appetite,Bitter taste of mouth,Abdominal distention,Headache,Loose stool,Nausea,Slippery pulse,Rapid pulse,White coating,Thick coating,Pale red tongue,Moderate pulse,Slimy coating,Sunken pulse,String-like pulse,Weak pulse,Teeth-marked tongue,Fine pulse,Rough pulse,Absence of thirst,Cough,Red tongue | Red tongue | 7% | Retained dampness toxin |
| P+S5_15 | Insomnia,Dry mouth,Lack of strength,Dizziness,Loss of appetite,Bitter taste of mouth,Abdominal distention,Headache,Loose stool,Nausea,Slippery pulse,Rapid pulse,White coating,Thick coating,Pale red tongue,Moderate pulse,Slimy coating,Sunken pulse,String-like pulse,Weak pulse,Teeth-marked tongue,Fine pulse,Rough pulse,Absence of thirst,Cough,Red tongue,Lumbago | Lumbago | 6% | Liver depression and spleen deficiency |
| P+S5_16 | Insomnia,Dry mouth,Lack of strength,Dizziness,Loss of appetite,Bitter taste of mouth,Abdominal distention,Headache,Loose stool,Nausea,Slippery pulse,Rapid pulse,White coating,Thick coating,Pale red tongue,Moderate pulse,Slimy coating,Sunken pulse,String-like pulse,Weak pulse,Teeth-marked tongue,Fine pulse,Rough pulse,Absence of thirst,Cough,Red tongue,Lumbago,Backache | Backache | 6% | Qi deficiency with blood stasis |
| P+S5_17 | Insomnia,Dry mouth,Lack of strength,Dizziness,Loss of appetite,Bitter taste of mouth,Abdominal distention,Headache,Loose stool,Nausea,Slippery pulse,Rapid pulse,White coating,Thick coating,Pale red tongue,Moderate pulse,Slimy coating,Sunken pulse,String-like pulse,Weak pulse,Teeth-marked tongue,Fine pulse,Rough pulse,Absence of thirst,Cough,Red tongue,Lumbago,Backache,Afraid of cold | Afraid of cold | 6% | Qi deficiency with blood stasis |
