# Supplementary material for: Application of Neural Network and Cluster Analyses to Differentiate TCM Patterns in Patients With Breast Cancer
Source: Front Pharmacol. 2020 May 8;11:670. doi: 10.3389/fphar.2020.00670 (PMC7227602; doi:10.3389/fphar.2020.00670)
Supplement: Supplementary file 2 [file Table_2.docx]

| **Supplementary Table 2** The definitions/descriptions from WHO of 8 main TCM patterns in breast cancer patients. | | |
| --- | --- | --- |
| **TCM pattern** | **Abbreviation** | **Definition/Description** |
| Liver-gallbladder dampness-heat | LGDH | Accumulation of dampness-heat in the liver and gallbladder resulting in impaired bile flow and downward pouring of dampness-heat.  **Liver**: the organ located in the right hypochondrium below the diaphragm, which stores blood, facilitates the coursing of qi, and is closely related to the function of the sinews and eyes.  **Gallbladder**: one of the six bowels, which, connecting with the liver, stores and discharges bile  **Dampness**: as a pathogenic factor characterized by its impediment to qi movement and its turbidity, heaviness, stickiness and downward flowing properties, also called pathogenic dampness.  **Heat**: heat as a pathogenic factor that causes heat pattern/syndrome, also called pathogenic heat. |
| Depressed liver qi transforming into fire | DLTF | A pattern/syndrome marked by distension, pain and burning sensation in the hypochondriac region, irritability, irascibility, bitterness and dryness in the mouth, reddened tongue with yellow coating and rapid string-like pulse, the same as the pattern/syndrome of stagnated liver qi transforming into fire.  **Liver qi**: essential qi of the liver, the physical substrata and dynamic force of the functional activities of the liver.  **Liver qi depression**: stagnation of qi in the liver resulting from impairment of free coursing, also called liver depression.  **Liver fire**: a pathological change of exuberant liver qi with heat manifestations. |
| Retained dampness-toxin/Retained dampness-heat | RDT/RDH | A pattern/syndrome marked by redness, swelling, ulceration and exudation of the hand, foot, ear, nose, head, face or genital region, or by fever, jaundice, impaired consciousness, eruptions, reddened tongue and rapid soggy pulse.  **Toxin**: any virulent pathogen that causes a fulminating disease. |
| Liver-kidney yin deficiency | LKYD | A pattern/syndrome attributed to insufficiency of yin fluid of the liver and the kidney with harassment of endogenous heat, marked by dizziness, blurred vision, tinnitus, forgetfulness, insomnia and dream-disturbed sleep, hypochondriac pain, aching lumbus and poor muscle tone in legs, flushed cheeks, heat sensation in the chest, palms and soles, night sweating, nocturnal emission in men and scant menstruation in women, reddened tongue with scanty coating, and rapid fine pulse.  **Liver yin**: the opposite of liver yang, the liver essence-blood and the quiescent and nutritive aspect of liver function, which also inhibits over activity of liver yang.  **Kidney yin**: the yin aspect of the kidney, which has a moistening, nourishing and cooling effect on all organs. |
| Liver depression and spleen deficiency | LDSD | a pattern/syndrome marked by hypochondriac and abdominal painful distension, depressed mood, frequent sighing, anorexia, uncomfortable loose bowels or alleviation of abdominal pain after defecation, borborygmi with flatus and white slimy tongue coating, the same as the pattern/syndrome of liver stagnation and spleen deficiency, also known as the liver-spleen disharmony pattern/syndrome.  **Spleen**: the organ located in the middle energizer below the diaphragm, whose main function is to transport and transform food, upbear the clear substances, keep the blood flowing within the vessels, and is closely related to the limbs and flesh. |
| Spleen-stomach qi deficiency | SSQD | A pathological change characterized by qi deficiency with impaired transporting and transforming function of the spleen and stomach with impaired appetite and digestion.  **Spleen qi**: essential qi of the spleen, the physical substrata and dynamic force of the functional activities of the spleen.  **Stomach qi**: essential qi of the stomach, the physical substrata and dynamic force of the functional activities of the stomach. Also used to denote a state of basic vitality detected by examination of the radial pulse. |
| Qi deficiency with blood stasis | QDBS | A pathological change of qi deficiency in which qi is insufficient to maintain blood flow, thus resulting in blood stasis.  **Qi deficiency**: a general term for deficiency of qi that leads to decreased visceral functions and lowered body resistance.  **Blood stasis**: a morbid state of blood stagnancy in a certain area of the body caused by sluggish flow of qi, deficiency of qi or blood, trauma, or yin-cold. |
