# Supplementary material for: Application of Neural Network and Cluster Analyses to Differentiate TCM Patterns in Patients With Breast Cancer
Source: Front Pharmacol. 2020 May 8;11:670. doi: 10.3389/fphar.2020.00670 (PMC7227602; doi:10.3389/fphar.2020.00670)
Supplement: Supplementary file 3 [file Table_3.docx]

| **Supplementary Table 3** Components of Chinese herbal formulas | |
| --- | --- |
| **Formula** | **Components** |
| Jia-Wei-Xiao-Yao-San | *Angelica sinensis* (Oliv.) Diels*, Poria cocos* (Schw.) Wolf*, Gardenia jasminoides* Ellis*, Mentha haplocalyx* Briq.*, Paeonia lactiflora* Pall.*, Bupleurum chinense* DC.*, Glycyrrhiza uralensis* Fisch.*, Atractylodes macrocephala* Koidz.*, Paeonia suffruticosa* Andr.*, Zingiber officinale* (Willd.) Rosc. |
| San-Zhong-Kui-Jian-Tang | *Scutellaria baicalensis* Georgi*, Gentiana manshurica* Kitag.*, Trichosanthes kirilowii* Maxim*., Phellodendron chinense* Schneid.*, Anemarrhena asphodeloides* Bge.*, Platycodon grandiflorum* (Jacq.) A. DC.*, Laminaria japonica* Aresch.*, Bupleurum chinense* DC.*, Glycyrrhiza uralensis* Fisch.*, Scirpus fluviatilis* (Torr.) A. Gray.*, Curcuma phaeocaulis* Val.*, Forsythia suspensa* (Thunb.) Vahl*, Pueraria thomsonii* Benth.*, Paeonia lactiflora* Pall.*, Angelica sinensis* (Oliv.) Diels*, Coptis chinensis* Franch.*, Cimicifuga foetida* L. |
| Xue-Fu-Zhu-Yu-Tang | *Angelica sinensis* (Oliv.) Diels*, Rehmannia glutinosa* Libosch.*, Prunus persica* (L.) Batsch*, Carthamus tinctorius* L.*, Citrus aurantium* L.*, Paeonia lactiflora* Pall.*, Bupleurum chinense* DC.*, Glycyrrhiza uralensis* Fisch.*, Platycodon grandiflorum* (Jacq.) A. DC.*, Ligusticum chuanxiong* Hort.*, Achyranthes bidentata* Bl. |
| Xiang-Sha-Liu-Jun-Zi-Tang | *Panax ginseng* C. A. Mey.*, Pinellia ternata* (Thunb.) Breit.*, Atractylodes macrocephala* Koidz.*, Poria cocos* (Schw.) Wolf*, Aucklandia lappa* Decne.*, Citrus reticulata* Blanco*, Amomum villosum* Lour.*, Glycyrrhiza uralensis* Fisch.*, Zingiber officinale* (Willd.) Rosc. |
| Gui-Pi-Tang | *Panax ginseng* C. A. Mey.*, Astragalus membranaceus* (Fisch.) Bunge*, Atractylodes macrocephala* Koidz.*, Poria cocos* (Schw.) Wolf*, Angelica sinensis* (Oliv.) Diels*, Ziziphus jujuba* Mill. var. *spinosa, Dimocarpus longan* Lour.*, Polygala tenuifolia* Willd.*, Aucklandia lappa* Decne.*, Glycyrrhiza uralensis* Fisch.*, Zingiber officinale* (Willd.) Rosc.*, Ziziphus jujuba* Mill. |
| Bu-Zhong-Yi-Qi-Tang | *Astragalus membranaceus* (Fisch.) Bunge*, Panax ginseng* C. A. Mey.*, Atractylodes macrocephala* Koidz.*, Glycyrrhiza uralensis* Fisch.*, Angelica sinensis* (Oliv.) Diels*, Citrus reticulata* Blanco*, Cimicifuga foetida* L.*, Bupleurum chinense* DC.*, Zingiber officinale* (Willd.) Rosc.*, Ziziphus jujuba* Mill. |
| Suan-Zao-Ren-Tang | *Ziziphus jujuba* Mill. var. *spinosa, Anemarrhena asphodeloides* Bge.*, Ligusticum chuanxiong* Hort.*, Poria cocos* (Schw.) Wolf*, Glycyrrhiza uralensis* Fisch. |
| Zhen-Ren-Huo-Ming-Yin | *Lonicera japonica* Thunb.*, Citrus reticulata* Blanco*, Angelica sinensis* (Oliv.) Diels*, Saposhnikovia divaricata* (Turcz.) Schischk.*, Angelica dahurica* Bentham et Hooker*, Glycyrrhiza uralensis* Fisch.*, Fritillaria thunbergii* Miq.*, Trichosanthes kirilowii* Maxim.*, Boswellia carterii* Birdw.*, Commiphora myrrha* Arn.*, Gleditsia sinensis* Lam. |
| Zhi-Bai-Di-Huang-Wan | *Rehmannia glutinosa* Libosch.*, Cornus officinalis* Sieb. et Zucc*., Poria cocos* (Schw.) Wolf*, Dioscorea opposita* Thunb.*, Paeonia suffruticosa* Andr.*, Alisma orientalis* (Sam.) Juzep.*, Anemarrhena asphodeloides* Bge.*, Phellodendron chinense* Schneid. |
| Sheng-Mai-Yin | *Panax ginseng* C. A. Mey.*, Ophiopogon japonicus* (Thunb.) Ker-Gawl.*, Schisandra sphenanthera* Rehd. et Wils. |
