# Supplementary material for: Application of Neural Network and Cluster Analyses to Differentiate TCM Patterns in Patients With Breast Cancer
Source: Front Pharmacol. 2020 May 8;11:670. doi: 10.3389/fphar.2020.00670 (PMC7227602; doi:10.3389/fphar.2020.00670)
Supplement: Supplementary file 4 [file Image_1.pdf]

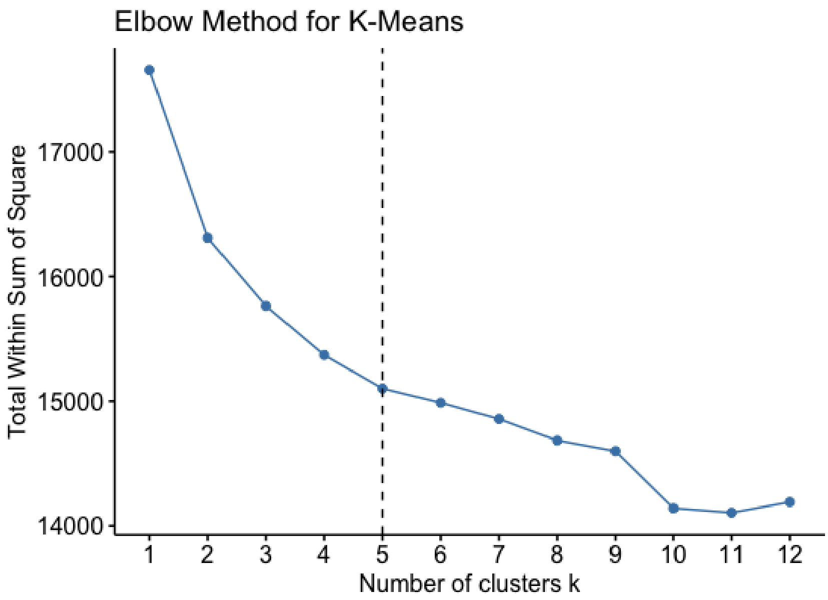

Supplementary Fig. 1 K-means clustering: the change of total within sum of square with different number of clusters.
